# Special Issue “Advances in Neurodegenerative Diseases Research and Therapy—Third Edition”

**DOI:** 10.3390/ijms27188267

**Published:** 2026-09-17

**Authors:** Sumonto Mitra

**Affiliations:** Division of Clinical Geriatrics, Center for Alzheimer Research, Department of NVS, Karolinska Institutet, 141 52 Huddinge, Sweden; sumonto.mitra@ki.se

Neurodegenerative diseases remain among the most pressing biomedical challenges of the twenty-first century. Despite decades of research, disorders such as Alzheimer’s disease (AD), Parkinson’s disease (PD), progressive supranuclear palsy (PSP), multiple sclerosis (MS), Huntington disease (HD) and related neurodegenerative conditions continue to affect millions of individuals worldwide, while effective disease-modifying therapies remain limited. Traditionally, these disorders have been defined by distinct pathological hallmarks, including amyloid-β and tau aggregates in AD and α-synuclein inclusions in PD and related synucleinopathies. However, growing evidence suggests that these pathological signatures represent only one dimension of a far more complex biological landscape involving cellular homeostasis, neuroimmune regulation, environmental exposures, and systemic biological interactions [1,2,3,4,5].

There is a growing recognition that disruptions of intracellular homeostasis represent common drivers of neurodegenerative disease. Increasing evidence implicates autophagy, lysosomal degradation, protein trafficking, mitochondrial maintenance, and endoplasmic reticulum (ER) function as central determinants of neuronal health [4,5]. The study by Martín-Rico et al. (Contribution 1), investigating Herpes simplex virus type 1 (HSV-1) infection in human 2D and 3D LUHMES in vitro neuronal models, exemplifies this concept by demonstrating that viral infection induces intracellular amyloid accumulation, tau hyperphosphorylation, and impairment of the autophagy–lysosome pathway. These observations not only provide mechanistic support for environmental contributions to neurodegeneration but also highlight the vulnerability of neuronal clearance mechanisms to external biological stressors.

Equally important is the recognition that neurodegeneration is fundamentally a multicellular process. While early disease models focused primarily on neuronal dysfunction, contemporary research has increasingly demonstrated the participation of astrocytes, microglia, oligodendrocytes, vascular cells, and peripheral immune systems in disease progression [6,7,8]. The imaging-transcriptomic study by Brusini et al. (Contribution 2) illustrates this paradigm by linking amyloid-β and tau-associated cortical remodelling to gene-expression profiles enriched in immune pathways and glial cell signatures among AD patients. The identification of astrocytes, microglia, and oligodendrocyte precursor cells as key biological correlates of structural brain alterations further supports the concept that neurodegeneration emerges from coordinated dysfunction across multiple cell populations rather than neuronal pathology alone.

Complementing these findings, the analysis by Vos et al. (Contribution 3) of reduced penetrance in Pink1-mutant Drosophila identifies ER-associated proteins as modifiers of disease expression, emphasizing that cellular stress-response pathways can actively influence disease outcomes. Collectively, these studies reinforce a growing consensus that failures in proteostasis and intracellular quality-control networks constitute a common biological foundation across seemingly distinct neurodegenerative disorders. Similarly, Pietrafesa et al. (Contribution 4) reported the molecular dynamics investigation of the Parkinson’s disease-associated genetic mutation in GBA1 E326K variant and demonstrates how subtle alterations in lysosomal protein interactions may have consequences that extend far beyond enzymatic activity alone. By affecting interactions with α-synuclein, saposin C, and LIMP-2, this study highlights the importance of intracellular trafficking and lysosomal organization in disease pathogenesis. This perspective is further strengthened by the review examining the genetic background of inflammatory mechanisms in PSP (Alster and Madetko-Alster (Contribution 5)). Although PSP has historically been considered primarily a tauopathy, accumulating evidence suggests that microglial activation and immune-related pathways may play central roles in disease susceptibility and progression.

Increasingly, neurodegenerative diseases are recognized as the product of complex interactions between genetic susceptibility and environmental exposures. This concept is exemplified by the HSV-1 study (Martín-Rico et al.), which highlights the potential consequences of viral infection on neuronal homeostasis and modifying AD pathological markers, and by the comprehensive review exploring the role of the gut microbiota in atypical Parkinsonian syndromes (Przewodowska et al. (Contribution 6)). Through its effects on immune regulation, metabolism, vitamin synthesis, and gut–brain communication, the intestinal microbiome has emerged as a potentially important modulator of neurodegenerative processes. These observations are consistent with growing evidence that the gut–brain axis contributes to neurological disease through bidirectional interactions involving microbial metabolites, inflammatory signalling, and neural pathways [9,10].

The review by Manna et al. (Contribution 7) addressing epigenetic mechanisms in MS further expands this framework by emphasizing the importance of gene–environment interactions. Epigenetic regulation may provide a mechanistic bridge between inherited susceptibility and environmental influences, contributing to disease heterogeneity and progression. Together, these contributions suggest that future models of neurodegeneration must account not only for central nervous system pathology but also for the cumulative influence of infectious exposures, microbiome dynamics, immune responses, metabolism, lifestyle, and aging. In this context, the concept of an individual’s neurological exposome may offer a useful framework for understanding disease risk across the lifespan.

Historically, neurodegenerative disease research has focused on identifying mechanisms that drive neuronal injury and death. However, an equally important question is increasingly gaining prominence: why do certain individuals or neuronal populations remain resistant despite substantial pathological burden? Understanding resilience may ultimately prove as important as understanding disease itself.

The study by Palasz et al. (Contribution 8) demonstrating that moderate aerobic exercise confers neuroprotection in an MPTP model of Parkinson’s disease provides an important example. By preserving dopaminergic neurons, enhancing BDNF and GDNF levels, and suppressing inflammation, moderate exercise produced robust neuroprotective effects comparable to those observed following more intense training paradigms. These findings are particularly encouraging because they suggest that clinically feasible interventions can meaningfully influence disease-relevant biological pathways. Likewise, the identification of ER-associated modifiers that reduce phenotypic expression in Pink1-deficient flies illustrates that endogenous protective mechanisms can substantially alter disease outcomes. Together, these studies shift attention from pathology alone toward a more balanced understanding of vulnerability and resilience. Another review by Olmedo-Saura et al. (Contribution 9) represents the current landscape of the various aspects of available treatments and clinical considerations for HD, including physical interventions and pharmacological approaches.

Future therapeutic strategies may increasingly focus on enhancing resilience mechanisms rather than exclusively attempting to eliminate pathological proteins. Neurotrophic signalling, metabolic adaptation, immune homeostasis, stress resistance, and adaptive network plasticity may emerge as critical therapeutic targets. In the coming years, biomarkers of resilience may become as valuable as biomarkers of pathology, enabling earlier and more individualized interventions aimed at preserving neuronal function before irreversible degeneration occurs.

Beyond their biological insights, the studies in this Special Issue collectively highlight the technological transformation currently reshaping neuroscience. Human-relevant 3D neuronal cultures, imaging transcriptomics, computational modelling, molecular dynamics simulations, and systems-level analyses are providing unprecedented opportunities to investigate disease mechanisms across multiple scales of biological organization. The 3D LUHMES platform for studying virus-induced neurodegeneration, the integration of neuroimaging and transcriptomics to characterize AD-related cortical remodelling, and the computational interrogation of Parkinson’s disease-associated mutations all exemplify how technological innovation is accelerating mechanistic discovery. Distinct diseases may ultimately be stratified according to shared patterns of proteostatic dysfunction, neuroimmune activation, environmental exposure, or resilience. Such a framework offers the potential to transform diagnosis, improve patient stratification, and facilitate the development of targeted disease-modifying therapies.

The contributions assembled in “Advances in Neurodegenerative Diseases Research and Therapy—Third Edition” collectively illustrate a major conceptual shift occurring across the field. Rather than viewing neurodegenerative diseases as isolated clinical entities, the studies included in this Special Issue reveal convergence across multiple biological networks that regulate neuronal vulnerability and resilience. Together, they support an emerging systems-level framework in which neurodegeneration is understood as the consequence of dynamic interactions among various processes. Simultaneously, growing recognition of resilience mechanisms is shifting the focus from why neurons die toward how they can be protected. The studies assembled in this Special Issue contribute meaningfully to this evolution and underscore the exciting opportunities that lie ahead for neurodegenerative disease research and therapy.
